# Molecular Cloning and Characterization of a Newly Isolated Pyrethroid-Degrading Esterase Gene from a Genomic Library of *Ochrobactrum anthropi* YZ-1

**DOI:** 10.1371/journal.pone.0077329

**Published:** 2013-10-14

**Authors:** Zhiyong Ruan, Yi Zhai, Jinlong Song, Yanhua Shi, Kang Li, Bin Zhao, Yanchun Yan

**Affiliations:** 1 College of Life Science and Technology, Huazhong Agricultural University, Wuhan, China; 2 Graduate School, Chinese Academy of Agricultural Science, Beijing, China; 3 Agricultural Engineering Institute, Chongqing Academy of Agricultural Sciences, Chongqing, China; 4 Institute of Agricultural Resources and Regional Planning, Chinese Academy of Agricultural Science, Beijing, China; 5 Institute for Biological Product Control, National Institutes for Food and Drug Control, Beijing, China; Universidad Autónoma del estado de Morelos, Mexico

## Abstract

A novel pyrethroid-degrading esterase gene *pytY* was isolated from the genomic library of *Ochrobactrum anthropi* YZ-1. It possesses an open reading frame (ORF) of 897 bp. Blast search showed that its deduced amino acid sequence shares moderate identities (30% to 46%) with most homologous esterases. Phylogenetic analysis revealed that PytY is a member of the esterase VI family. *pytY* showed very low sequence similarity compared with reported pyrethroid-degrading genes. PytY was expressed, purified, and characterized. Enzyme assay revealed that PytY is a broad-spectrum degrading enzyme that can degrade various pyrethroids. It is a new pyrethroid-degrading gene and enriches genetic resource. Kinetic constants of *K*m and Vmax were 2.34 mmol·L^−1^ and 56.33 nmol min^−1^, respectively, with lambda-cyhalothrin as substrate. PytY displayed good degrading ability and stability over a broad range of temperature and pH. The optimal temperature and pH were of 35°C and 7.5. No cofactors were required for enzyme activity. The results highlighted the potential use of PytY in the elimination of pyrethroid residuals from contaminated environments.

## Introduction

Pyrethroids are globally used pesticides. They play an important role in control of agriculture, forestry, and indoor pests. Pyrethroids are a large class of synthetic compounds based on the structure of pyrethrin, which is a natural insecticidal toxin from *Chrysanthemum cinerariaefolium* flowers [1]. Since the discovery and commercial production, pyrethroids have been used for more than 30 years and have accounted for almost 25% of the global pesticide market [2]. Pyrethroids possess high insecticidal activity. But, they are low toxicity to mammals and have been considered as ideal replacements for some highly toxic pesticides. Traditional organochlorine and organophosphorus pesticides have been phased out due to high toxicity and recalcitrant characteristics. Thereby, the demand for pyrethroid pesticides continues to increase [3].

Generally, pyrethroids are considered to be low toxicity. However, the widespread use of pyrethroids has caused increasing public concerns about human health risk and environmental issues, such as atmosphere, soil, and water pollution, agricultural products with high pesticide residues. Some studies have indicated that pyrethroids are not as safe as believed. Researchers found that pyrethroids can induce neurotoxicity, reproductive toxicity, cytotoxicity, and even the risk of mutation, teratogenicity, and carcinogenicity [4], [5], [6], [7]. Long-term exposure to pyrethroids may cause chronic diseases and do harm to many tissues [8], [9], [10]. Some pyrethroids have been classified as possible carcinogen to human by the US Environmental Protection Agency (EPA) [11], [2]. Additionally, pyrethroid pesticides also possess high acute toxicity to some non-target organisms, especially pest predators, fish, and aquatic invertebrates [12], [13]. These issues are closely related to our health and ecological safety. People are giving more and more attention to the residues and persistence of pyrethroids in the environment. Efficient methods must be developed to solve these problems caused by pyrethroids residues.

Biodegradation is an important biotechnology which mainly involves the application of living organisms to break down organic pollutants. It is generally considered as a safe, efficient, and inexpensive way to eliminate environmental contaminants [14]. In the natural environment, pyrethroids residues can be degraded by abiotic or biotic mode, including photolysis, hydrolysis, and biodegradation [15]. Microbial decomposition has a key function in the degradation of pyrethroid residuals in contaminated soil, sediment, and sewage treatment systems [16]. Many pyrethroid-degrading microorganisms have been isolated and studied. Most studies have focused on the determination of the degrading ability of strains, analysis of metabolites, and purification of some related degrading enzymes [8], [2], [17], [18], [19]. Reports about pyrethroid-degrading genes are rare. Pyrethroids possess similar chemical structure formed by an alcohol and acid moieties with an ester bond. The major metabolic pathway of pyrethroids in resistant insects and degrading microorganisms involves cytochrome P450 oxidation and ester-bond hydrolysis by esterase [20]. Esterase belongs to hydrolase and is capable of hydrolyzing a large number of ester-containing compounds, such as carbamates, organophosphorus pesticides, and pyrethroids [21], [22]. Some pyrethroid-degrading enzymes have been purified and characterized [23], [24], [25]. Compared with living microorganisms, the degrading gene has a greater potential in eliminating pyrethroid residuals, especially with mass production of degrading enzymes. To date, only a few pyrethroid-degrading genes, such as *estP*, *pytH*, *pye3*, and *pytZ*, were reported [26], [27], [28], [29].

In this work, a new pyrethroid-degrading gene *pytY* was isolated by screening the genomic library of *Ochrobactrum anthropi* YZ-1, and was expressed in *Escherichia coli* BL21(DE3). Properties of the purified degrading enzyme, such as substrate specificity, optimal temperature and pH, stability of temperature and pH, and kinetic parameters, were determined. In previous studies, all the isolated pyrethroid-degrading genes from genomic libraries or metagenomes have been single genes. *pytY* is the second degrading gene isolated from strain YZ-1. To our knowledge, prior to this work, no report has been conducted about two genes in the same strain both contributing to pyrethroids degradation.

## Materials and Methods

### Strains, Plasmids, Library, and Media

The used strains and vectors were of *E. coli* DH5α, *E. coli* BL21 (DE3) (Tiangen), pUC18 (Takara), and pET30a(+) (Novagen). The genomic library of *Ochrobactrum anthropi* YZ-1 was previously constructed and preserved in our laboratory. Mineral salt medium that containing 1.0 g·L^−1^ of NH_4_NO_3_, 0.5 g·L^−1^ of NaCl, 0.5 g·L^−1^ of (NH_4_)_2_SO_4_, 0.5 g·L^−1^ of KH_2_PO_4_, and 1.5 g·L^−1^ of K_2_HPO_4_ was used for library screening. Luria–Bertani medium that containing 10.0 g·L^−1^ of tryptone, 5.0 g·L^−1^ of yeast extract, and 10.0 g·L^−1^ of NaCl was used for recombinant protein expression. Different antibiotics were added corresponding to the vector resistance when required.

### Chemical Reagents and Enzymes

Technical-grade pyrethroids were provided by Jiangsu Yangnong Chemical Group Co., Ltd. Standards were purchased from the National Institute of Metrology, P.R. China. Fast-Blue RR Salt, *α*-naphthyl acetate, and other p-nitrophenyl esters were products of Sigma. *Sau*3AI, *Bam*HI, *Eco*RI, CIAP, and T4 DNA ligase were purchased from Takara. All other chemicals were of analytical grade. Different pyrethroids were dissolved in chromatographic-grade acetone at a final concentration of 1.0×10^5 ^mg·L^−1^, sterilized by membrane filtration, and placed in the dark as a stock solution.

### Screening of Genomic Library

Library screening was performed according to the method as detailed in previous report [29]. The inserted DNA fragment in the target clone was sent to Invitrogen Biotechnology Co., Ltd. for sequencing. The ORF was analyzed using molecular biology software DNAman 6.0 and ORF Finder online tool at http://www.ncbi.nlm.nih.gov/gorf/gorf.html. Then, the putative ORFs were subcloned into pET30a vector and used to transform *E. coli* BL21(DE3) to determine their pyrethroid-degrading ability.

### Sequence Analysis

For the target ORF of *pytY*, sequence similarity was analyzed by online server of Blast program at National Center of Biotechnology Information (NCBI) http://www.blast.ncbi.nlm.nih.gov/. Multiple sequence alignment was performed with Clustal W program [30] and identical amino acid residues were visually shaded using GeneDoc software. Up to 24 bacterial esterase/lipase sequences from eight different families were used to determine which esterase family PytY belongs to by neighbor-joining method using MEGA 4.0. Bootstrapping of 1,000 replicates was carried out to estimate the confidence levels of phylogenetic reconstructions [31].

### Expression and Purification of Recombinant Protein PytY

The complete ORF sequence of *pytY* was amplified by PCR using primers ZF (5′- CGGGATCCATGACCACTCAAACCTATGAGC-3′, *Bam*HI restriction site is underlined) and ZR (5′-CGGAATTCTCAGTATGCGAGAAGCGACTG -3′, *Eco*RI restriction site is underlined). The amplified product was inserted into the *Bam*HI-*Eco*RI site of pET30a (+) vector, resulting in the recombinant plasmid pET30a-*pytY*. The culture, induction, and harvest of recombinant *E. coli* BL21(DE3) cell carrying pET30a-*pytY* were performed according to the standard method [32]. Recombinant protein was purified using Ni-NTA Fast Start Kit (Qiagen) according to the product operating manual. The purified protein was analyzed by sodium dodecyl sulfate polyacrylamide gel electrophoresis (SDS–PAGE). Protein concentration was measured by the Bradford method with bovine albumin as the standard [33].

### Substrate Specificity and Kinetics Study

Lambda-cyhalothrin, beta-cypermethrin, beta-cyfluthrin, deltamethrin, and permethrin were tested to determine the substrate specificity. The hydrolytic activity of PytY to different pyrethroids was measured according to the method detailed previously [24] and represented as a relative activity of the optimal substrate. One activity unit was defined as the required amount of enzyme to hydrolyze 1 nmol substrate per minute.

Enzyme kinetics study was conducted using lambda-cyhalothrin as the optimal substrate. Lambda-cyhalothrin was set in six different concentrations for initial velocity determination. The kinetic constants were obtained by Lineweaver–Burk plots. For each test, no more than 10% of substrate was consumed during the assay, and solvent content never exceeded 1.0% of the total assay volume.

### Effects of Temperature and pH on the Enzymatic Activity

The effect of temperature on enzyme activity was investigated by incubating the purified PytY and lambda-cyhalothrin substrate in 50 mmol·L^−1^ PBS buffer (pH 7.5) at different temperatures (15°C to 60°C). The treatment without PytY was set as control. For thermostability determination, the sample was pre-incubated in 50 mmol·L^−1^ PBS buffer at different temperatures for 2 h. The remaining activity was determined and represented as a percentage of the activity without pre-incubation. Effect of pH on PytY was also tested at different pH buffers (pH 4.0 to 10): disodium phosphate-citric acid buffer (pH 4.0 to 7.0) and Tris-HCl (pH 7.5 to 10.0). The determination of pH stability was performed by pre-incubating PytY in a corresponding buffer for 2 h, and then the residual activity was measured.

### Effect of Metal Ions and Chemical Regents on Enzymatic Activity

To investigate the effect of some metal ions and chemical regents on enzymatic activity, purified PytY and lambda-cyhalothrin substrate were incubated in PBS buffer (pH 7.5) with various metal ions and chemical agents, such as Na^+^, K^+^, Mg^2+^, Zn^2+^, Fe^2+^, Ag^+^, and Hg^2+^ (1 mmol·L^−1^); Tween-20 and Tween-80 (1.0%); SDS, phenylmethylsulfonyl fluoride (PMSF) and diethyl pyrocarbonate (DEPC) (1 mmol·L^−1^); chelating agents ethylenediamine tetraacetic acid (EDTA) and 1,10-phenanthroline (1 mmol·L^−1^). The enzymatic activity was measured and expressed as a percentage of the activity of control treatment which was in the absence of metal ions or chemical regents.

### Gas Chromatography (GC) Analysis

Residual pyrethroid in reaction system was extracted twice with an equal volume n-hexane. The organic phase was filtrated and evaporated by vacuum freeze-drying. The residue was re-dissolved in n-hexane. Samples were analyzed by a GC-2010 system equipped with a RTX-1301 chromatographic column (30.0 m×0.25 mm×0.25 µm). The procedure was as follows: The mobile phase was of N_2_ (purity>99.999%) with a flow rate of 1.0 mL·min^−1^ and inlet temperature was set at 260°C. The temperature programming was 230°C for the initial 8 min, increased to 280°C at a velocity of 25°C ·min^−1^, and finally retained at the said temperature for 6 min. The injection volume was 1 µl with split ratio of 49∶1. An electron capture detector was used at the detection temperature of 300°C.

## Results

### Screening, Sequence and Phylogenetic Analysis of Pyrethroid-degrading Gene

**Figure 1:**
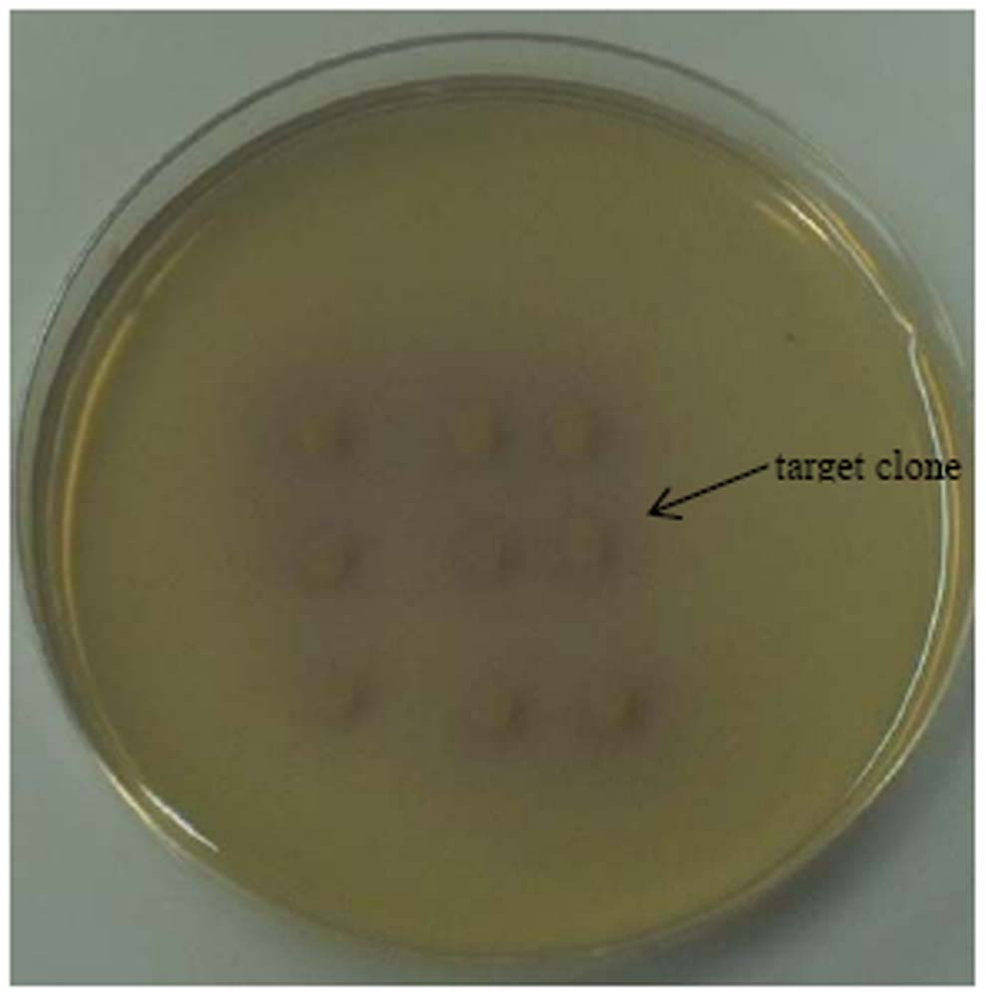
The initial screening of transformants. The obtained target clone is indicated by an arrow.

The screening result is shown in Fig. 1. One positive clone was obtained from the remaining genomic library of strain *Ochrobactrum anthropi* YZ-1. The collection number of this strain is CGMCC1.12044 in China General Microbiological Culture Collection Center. The inserted fragment in recombinant plasmid was 1340 bp and contained two putative ORFs. The two ORFs were then subcloned for functional determination. Only one ORF displayed degrading ability to pyrethroids and was proven to be a degrading gene. This ORF was designated as *pytY*, and the sequence was deposited in GenBank with the accession number JQ025998. The other ORF was not capable of degrading pyrethroids.

**Figure 2:**
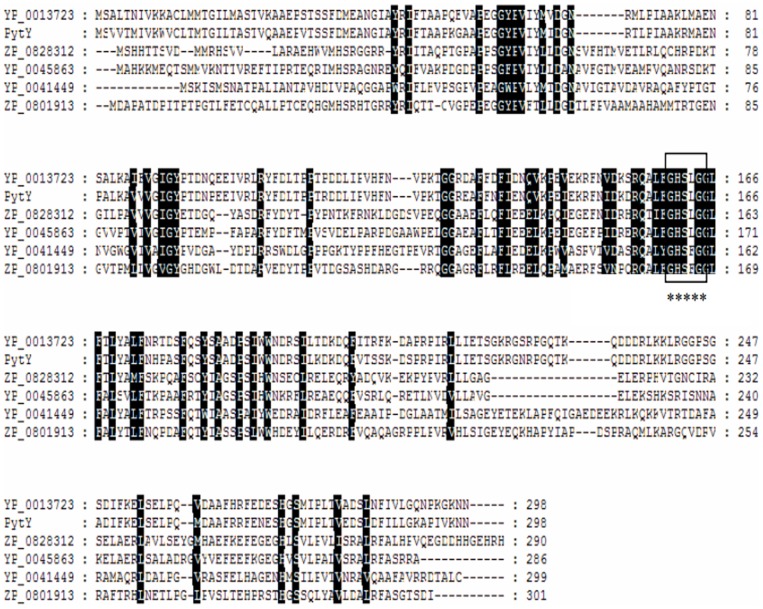
Multiple alignments of deduced amino acid sequences of PytY with other related proteins. The conserved motif of “Gly-X-Ser-X-Gly” is marked with a frame and “*”.

The deduced amino acid sequence of PytY showed moderate identity with some esterase sequences available in the NCBI database, such as putative esterase from *Acetonema longum* DSM 6540 (ZP_08622994.1, 46% identity), *Pectobacterium wasabiae* WPP 163 (YP_003258613.1, 36% identity), and *Azorhizobium caulinodans* ORS 571 (YP_001526166.1, 35% identity), except for a putative esterase from *Ochrobactrum anthropi* ATCC 49188 (YP_001372331.1, 85% identity), as shown in Fig. 2.

**Figure 3:**
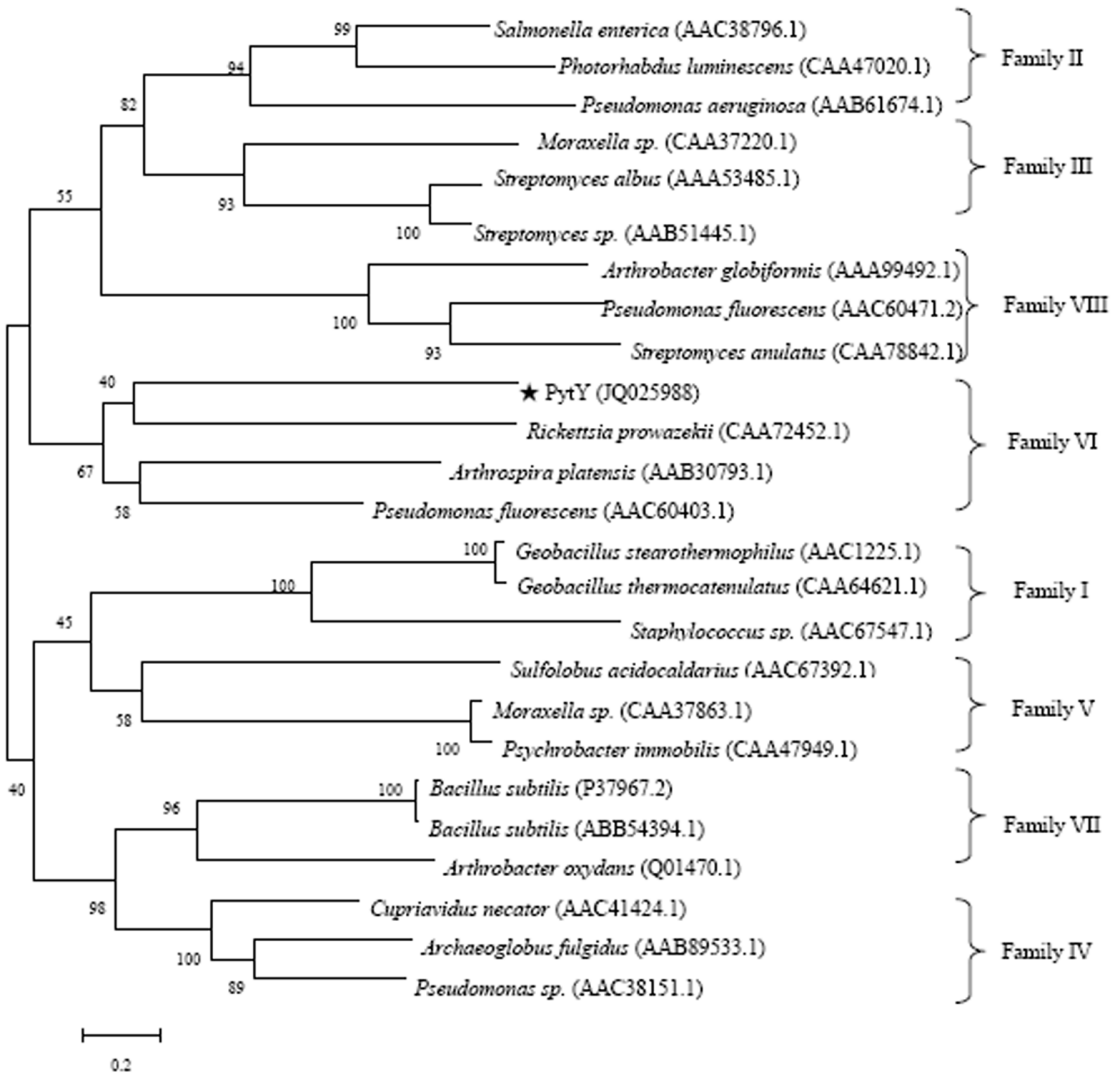
Phylogenetic relationship of PytY and proteins of eight different esterase families. The accession numbers of the protein sequences are in brackets.

Phylogenetic analysis was shown in Fig. 3. PytY was clustered with *Rickettsia prowazekii* (CAA72452.1), *Arthrospira platensis* (AAB30793.1), and *Pseudomonas fluorescens* (AAC60403.1), which belonged to the same esterase family VI.

### Expression and Purification of PytY

**Figure 4:**
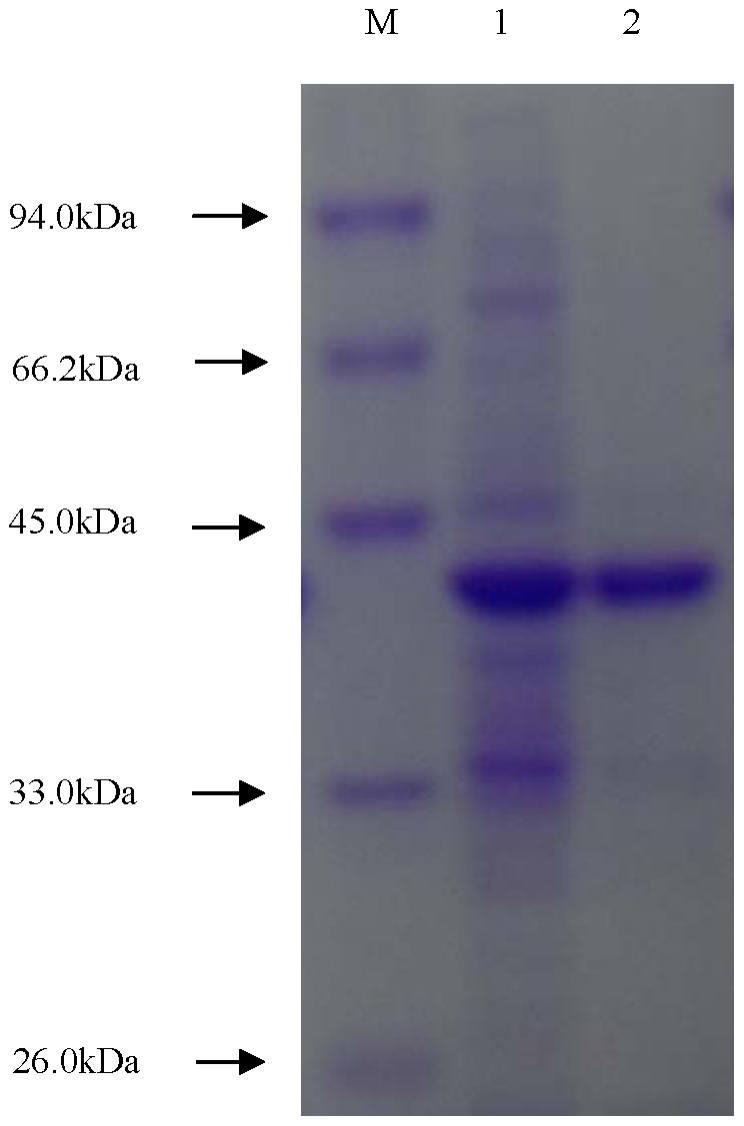
Expression, purification and SDS-PAGE of recombinant protein PytY. M: protein marker, lane 1: total protein of *E. coli* BL21 (DE3)/pET30a-*pytY*, lane 2: purified PytY protein.

For further functional study, recombinant protein PytY was expressed in *E. coli* BL21(DE3) with pET30a (+) vector. The purified recombinant protein was shown in Fig. 4. It gave a clear band of approximately 42 kDa on SDS–PAGE, in good agreement with the molecular mass of 41.7 kDa from the deduced amino acid sequence of PytY.

### Substrate Specificity and Kinetics Study

**Figure 5:**
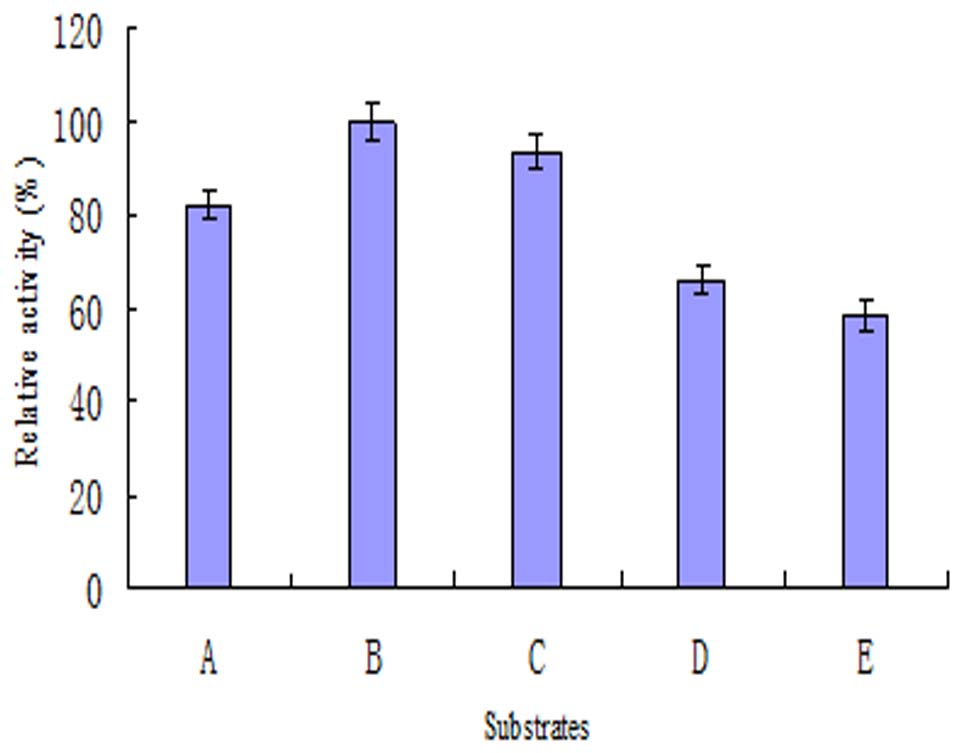
Substrate specificity analysis of purified PytY protein. A: beta- cyfluthrin, B: lambda-cyhalothrin, C: beta-cypermethrin, D: permethrin, E: deltamethrin. All the determinations were in triplicate.

Substrate specificity was determined using various pyrethroids. The result was shown in Fig. 5. PytY degraded all the tested substrates, suggesting that PytY was a broad-spectrum pyrethroid-hydrolyzing enzyme. However, PytY displayed different activities to different pyrethroids, among which lambda-cyhalothrin was proven to be the optimal substrate but deltamethrin was the most persistent.

Kinetic studies showed that when lambda-cyhalothrin was used as the substrate, kinetic constants of *K*_m_ and V_max_ were 2.34 mmol·L^−1^ and 56.33 nmol·min^−1^, respectively.

### Effect of Temperature and pH on the Enzyme Activity

**Figure 6:**
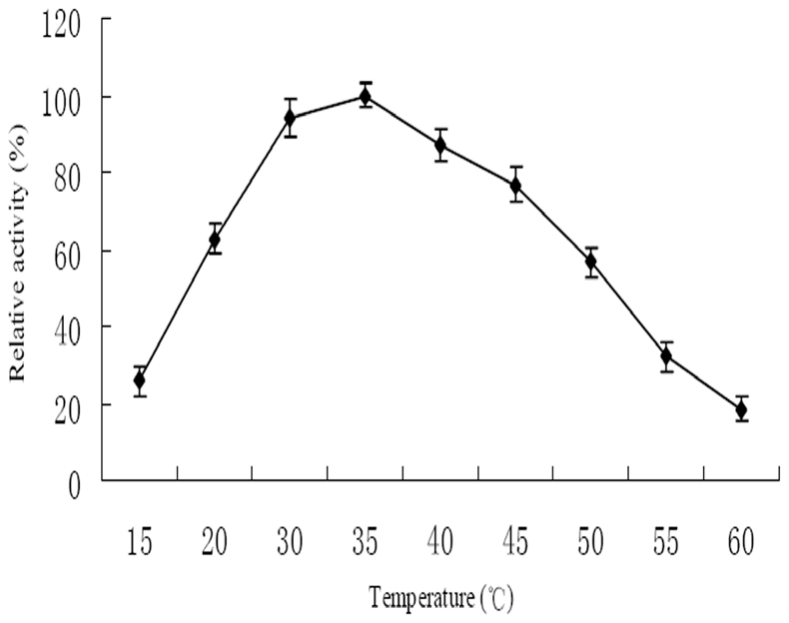
Effect of temperature on enzyme activity of purified PytY protein. The highest activity at 35°C was set as 100%. Each treatment was done in triplicate.

It could be seen from Fig. 6, the optimal temperature was 35°C, with the highest enzyme activity of 121 U. PytY displayed better degrading ability within the temperature range from 30°C to 45°C. The thermal stability showed that PytY was stable even when incubated for 2 h within the temperature range from 15°C to 45°C. When temperature was higher than 45°C, the stability began to decrease. The reason might be that higher temperature made part of enzyme inactivation.

**Figure 7:**
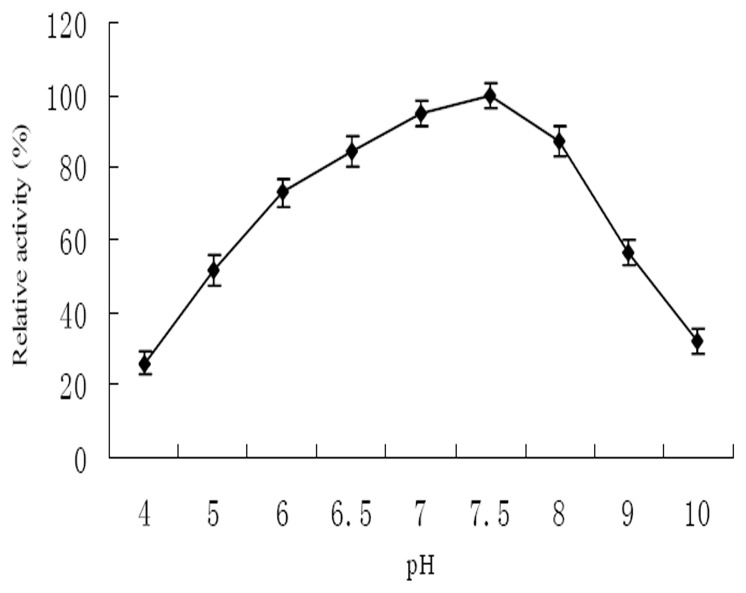
Effect of pH on enzyme activity of purified PytY protein. The highest activity at pH%. All measurements were done in triplicate.

The effect of pH on enzyme activity and stability of PytY was shown in Fig. 7. PytY showed higher enzyme activity within the pH range from 5.0 to 9.0, and the optimal pH was 7.5. PytY was stable within the pH range from 5.0 to 7.5. The remaining enzyme activity was more than 70% after the 2 h incubation.

### Effect of Metal Ions and Chemical Agents on the Enzyme Activity

As shown in Tables 1 and 2, the enzyme activity of PytY was strongly inhibited by Ag^+^, Hg^2+^, SDS, Ser protease inhibitor PMSF, and His modifier DEPC. Mg^2+^, surfactant Tween-20, and Tween-80 resulted in a slight increase of enzyme activity. The effect of chelating agents EDTA and 1,10-phenanthroline was not obvious compared with those of other tested compounds.

**Table 1:** Effects of metal ions on the enzyme activity of PytY.

Metal ions	Concentration (mM)	Relative activity (%)
Control	0	100.2±3.6
Na^+^	1.0	106.4±2.7
K^+^	1.0	96.3±2.5
Mg^2+^	1.0	112.6±4.2
Zn^2+^	1.0	92.4±2.4
Fe^2+^	1.0	103.2±3.6
Ag^+^	1.0	13.2±2.1
Hg^2+^	1.0	8.5±1.6

Enzyme activity without addition of metal ions was set as 100%.

**Table 2:** Effects of chemical reagents on the enzyme activity of PytY.

Chemical reagents	Concentration	Relative activity (%)
Control	0	100.0±2.4
Tween-20	1.0%	116.4±3.6
Tween-80	1.0%	112.4±4.5
SDS	1 mM	7.2±0.8
PMSF	1 mM	9.4±1.2
DEPC	0.1%	15.7±2.2
EDTA	1 mM	93.6±4.1
1,10-phenanthroline	1 mM	94.7±4.4

Enzyme activity without addition of chemical reagents was set as 100%.

## Discussion

Pyrethroids are a large class of ester-containing compounds whose main degradation route involves cleavage of the ester bond [34]. Previous study has proven that esterases detoxify pyrethroids by hydrolyzing the ester bond [22]. In the present study, the initial screening resulted in the detection of nine transformants based on the brown halo in the indicator medium, which are shown in Fig. 1. However, only one of them was proven to be the target clone in further screening, suggesting that not all the esterases are capable of degrading pyrethroids. So far, reported pyrethroid-degrading genes from genomic library are all single gene. However, *pytY* is the second degrading gene isolated from the strain of *O. anthropi* YZ-1. To our knowledge, this is the first report on two genes from the same strain and both contributing to pyrethroid degradation. To date, the screening of pyrethroid-degrading genes from the library mainly relies on indirect methods by determining degradation ability of the isolated genes [26], [27], [28], [29].

Sequence analysis indicated that *pytY* possessed a typical conserved domain of esterase/lipase superfamily. The deduced amino acid sequence of PytY showed moderate identity with some esterase sequences available in the NCBI database, such as putative esterase from *Acetonema longum* DSM 6540 (ZP_08622994.1, 46% identity), *Pectobacterium wasabiae* WPP 163 (YP_003258613.1, 36% identity), and *Azorhizobium caulinodans* ORS 571 (YP_001526166.1, 35% identity), except for a putative esterase from *Ochrobactrum anthropi* ATCC 49188 (YP_001372331.1, 85% identity). Multiple alignment revealed that PytY possesses the conserved pentapeptide motif of Gly-X-Ser-X-Gly [35]. In the center of the motif, the Ser^162^ residue acts as nucleophile that catalyzes the hydrolysis of ester bond [36], which is a typical feature of esterase. This result indicated that *pytY* was a pyrethroid-degrading esterase gene. In the phylogenetic analysis, PytY was clustered with *Rickettsia prowazekii* (CAA72452.1), *Arthrospira platensis* (AAB30793.1), and *Pseudomonas fluorescens* (AAC60403.1), which belonged to family VI [37], suggesting a new number of this family. *pytY* showed very low sequence similarity compared with other reported pyrethroid-degrading genes, even the *pytY* that was isolated from same strain *O.anthropi* YZ-1. Therefore, *pytY* is a new pyrethroid-degrading gene. It enriches the genetic resources for bioremediation of pyrethroid residues.

PytY was easily produced with the induction of 1 mmoL^−1^ IPTG at 30°C. It is a moderate pyrethroid-degrading enzyme, smaller than permethrinase (61 kDa) from *Bacillus cereus* SM3 [17], pyrethroid hydrolase (56 kDa) from *Aspergillus niger* ZD11 [18], carboxylesterase (60 kDa) from mouse liver microsomes [24], carboxylesterase E3 (58.6 kDa) from *Nephotettix cincticeps* Uhler [25], and EstP (73 kDa) from *Klebsiella* sp.ZD112 [26], but bigger than the carboxylesterase (31 kDa) from *Sphingobium* sp.JZ-1 [27], esterase (31.15 kDa) from the metagenome [28], and carboxylesterase PytZ (24.2 kDa) from *Ochrobactrum anthropi* YZ-1 [29]. Generally, inducible recombinant proteins tend to form inactive inclusion bodies when excessively expressed. That is not conductive for further study. In the present study, PytY protein was easily expressed and the induced recombinant protein was soluble, which facilitated its purification and functional studies. Moreover, this characteristic allows mass production of the recombinant protein for practical applications in the future.

PytY was capable of degrading all the tested substrates, suggesting that PytY was a broad-spectrum pyrethroid-hydrolyzing enzyme. This result may be related to the similar molecular structure of pyrethroid pesticides [34]. In the environment, pyrethroid residual is generally a mixture of multiple pyrethroids. Therefore, broad-spectrum degrading enzyme would have greater practical application in bioremediation. PytY displayed different activities to various pyrethroids. The reason might be that small differences among the structures of the substrates changed the activity of the enzyme. Additionally, *p*-nitrophenyl ester test revealed that PytY was an esterase, not a lipase (data not shown).

The Kinetic constants *K*_m_ and V_max_ of PytY are 2.34 mmol·L^−1^ and 56.33 nmol·min^−1^ respectively when lambda-cyhalothrin was used as the substrate. In previous study, the measured *K*_m_ and V_max_ of PytZ are 2.65 nmol·min^−1^ and 53.19 nmol·min^−1^ at the same conditions. This indicated that PytY possesses a higher degrading ability compared with PytZ.

Enzyme activity can be significantly influenced by temperature and pH. Each enzyme has a suitable temperature and pH range in which it efficiently works. In the present study, PytY displayed high activity and stability over a broad range of temperature and pH. These characteristics make PytY a potential candidate for eliminating pyrethroids residues.

The effect of chelating agents EDTA and 1, 10-phenanthroline on enzymatic activity was not obvious, which indicated that PytY might not require a cofactor during the hydrolysis of pyrethroids. Some Ser proteases usually form a lid in tertiary structures to prevent the inhibition of PMSF on Ser residues [38]. In this test, the enzyme activity of PytY was heavily inhibited by PMSF, suggesting that this pyrethroid-degrading esterase did not possess the lid structure. These characterizations were similar to pyrethroid hydrolase PytZ, which was previously reported [29].

In conclusion, another pyrethroid-degrading gene *pytY* was successfully cloned from the genomic library of *O. anthropi* YZ-1. This enriches pyrethroid-degrading gene resource. PytY displays a broad substrate spectrum, high enzyme activity, and favorable stability over a broader range of temperature and pH. These properties show that the pyrethroid-degrading enzyme PytY is a potential candidate for the detoxification of pyrethroid residues, taken the complexity of environment into consideration. In environmental biotechnology, it can be applied to bioremediation in pyrethroids residues degradation. Further studies about this degrading enzyme are in the works.
